# Vonoprazan-related adverse events: a pharmacovigilance study based on the FDA adverse event reporting system

**DOI:** 10.3389/fmed.2025.1688914

**Published:** 2025-12-19

**Authors:** Yali Zhong, Cong Hou, Jie Zhang, Li Wang, Dongmei Xiong, Huang Zhong, Xiaobin Luo

**Affiliations:** 1 Department of Gastroenterology, Zigong First People’s Hospital, Zigong, Sichuan, China; 2 Department of Neurosurgery, Zigong Fourth People’s Hospital, Zigong, Sichuan, China; 3 Department of Neurosurgery, The Affiliated Hospital of Southwest Medical University, Luzhou, Sichuan, China; 4 Sichuan Provincial Neurosurgery Clinical Medical Research Center, Luzhou, Sichuan, China

**Keywords:** Vonoprazan, adverse events, FAERS, signal detection, TTO, WSP

## Abstract

This study comprehensively evaluated the safety profile of Vonoprazan using data from the FDA Adverse Event Reporting System (FAERS) from the first quarter (Q1) of 2023 through the first quarter (Q1) of 2025. This analysis was restricted to real-world reports collected after the U. S. approval of vonoprazan, to better reflect its current clinical use pattern in the United States. Reports in which Vonoprazan was designated as the primary suspect(PS) drug were systematically extracted, and duplicate entries were removed using MySQL(version 8.0.42). Adverse events were coded in according tothe Medical Dictionary for Regulatory Activities (MedDRA, version 24.0). To detect potential safety signals, four disproportionality methods were applied: Reporting Odds Ratio (ROR), Proportional Reporting Ratio (PRR), Bayesian Confidence Propagation Neural Network (BCPNN), and Multi-Item Gamma Poisson Shrinker (MGPS). Differences between serious and non-serious adverse events were further examined using Pearson’s chi-square test and Fisher’s exact test. Temporal patterns of adverse event occurrence were evaluated using Time-to-Onset (TTO) analysis and Weibull Shape Parameter (WSP) modeling. In total, 978 vonoprazan-related cases were included; females accounted 62.0% of reports, and 34.3% of events were classified as serious. Forty seven preferred terms (PTs) met predefined signal criteria, and 16 of these, such as facial paralysis and cholecystitis, were not listed in the current U. S. FDA prescribing information for vonoprazan. Serious adverse events were more often associated with renal impairment and haematemesis, whereas non-serious events were predominantly gastrointestinal. Stratified analyses further revealed several potential high-risk signals, particularly inmale and elderly patients. The median time to onset was 7 days, and most events occurred within the first 30 days after treatment initiation. Weighted Survival Probability (WSP) analysis suggested an “early failure” pattern, and co-administration of aspirin and other acid-suppressing agents was common. In conclusion, this study provides a systematic characterization of Vonoprazan- associated adverse events and highlights several potential new safety signals that are not currently reflected in the drug’s labeling, these findings should be interpreted as hypothesis-generating safety signals that may inform future prospective monitoring and clinician counseling.

## 1 Introduction

Vonoprazan is a novel potassium-competitive acid blocker (P-CAB) that provides more potent and sustained gastric acid suppression when compared to conventional proton pump inhibitors (PPIs) (1). It demonstrates notable efficacy in relieving heartburn associated with esophagitis and gastroesophageal reflux disease (GERD), outperforming PPI formulations and gaining wide clinical acceptance (2). Initially developed and launched in Japan in 2014, vonoprazan received marketing authorization in 2015 for the treatment of acid-related disorders (3). On May 3, 2022, the U. S. FDA approved vonoprazan-containing regimens-Voquezna Dual Pak and Voquezna Triple Pak- for the eradication of *Helicobacter pylori* infections in adults. Subsequently, on November 1, 2023, Vonoprazan received its first U. S. FDA approval as a monotherapy for the treatment of Celiac disease and Gastroesophageal Reflux Disease (GERD). Furthermore, on July 18, 2024, the FDA granted an additional indication for Vonoprazan for the management of heartburn symptoms associated with non-erosive GERD in adults (4).

Vonoprazan has demonstrated a favorable safety and efficacy profile in the management of erosive esophagitis (ERD), effectively mitigating nocturnal acid breakthrough and alleviating reflux symptoms in patients unresponsive to standard treatments (5). It has also shown promising outcomes in the treatment of non-erosive esophagitis (NERD), particularly in patients who exhibit suboptimal responses to conventional proton pump inhibitors (PPIs) (6, 7). In *Helicobacter pylori* eradication therapy, Vonoprazan surpasses traditional PPI-base regimens in both first-line and salvage therapy, especially among patients with clarithromycin resistance. Vonoprazan demonstrates significantly greater efficacy compared to proton pump inhibitors (PPIs) (8). Furthermore, dual therapy with Vonoprazan and amoxicillin has the potential to replace conventional triple therapy in certain cases, offering a reduction in side effects (8). In terms of safety, Vonoprazan is generally well tolerated, demonstrating a safety profile comparable to that of PPIsand a lower incidence of adverse events (1). In addition, Vonoprazan has shownenhanced efficacy and safety when combined with other agents such as amoxicillin, particularly against clarithromycin- resistant *H. pylori* strains (1). The efficacy of Vonoprazan in the eradication of *H. pylori* is notable, positioning it as the most effective pharmacological treatment regimen currently available (9).

Lin et al. (10) investigated the efficacy and tolerability of Vonoprazan combined with varying doses of amoxicillin for *H. pylori* eradication. Reported adverse effects included abdominal bloating (12 cases), nausea and vomiting (11 cases), abdominal pain (7 cases), and acid reflux (7 csses). No significant associations were observed between these adverse effects and demographic variables such as height, weight, or gender. A small subset of patients also reported influenza-like symptoms, including mild chest pain and generalized myalgia. Moreover, patients receiving Vonoprazan exhibited elevated serum gastrin levels, which may inducehyperplasia of enterochromaffin-like (ECL) cells in the gastric mucosa and potentially increasing the long-term risk of gastrointestinal neoplasia (11). Prolonged acid suppression may also alter the intestinal microbiota, predisposing patients to Clostridioides difficile infections (12). In addition, long-term therapy may cause nutrient malabsorption, leading to secondary anemia, hypomagnesemia, and hypocalcemia (13). There is also evidence to suggesting possible associations with nephritis, pneumonia, and ischemic cardiac disorders (14, 15).

Vonoprazan is primarily metabolized in the liver via cytochrome P450 enzymes, particularly CYP3A4 and CYP3A5 (16). Its excretion occurs mainly through the renal pathway, being eliminated via urine and feces. Kong et al. developed a physiologically based pharmacokinetic-pharmacodynamic (PBPK-PD) model to investigate the pharmacokinetic characteristics and acid-suppressive effects of vonoprazan in rats, dogs, and humans. The results showed that both single and multiple dosing of vonoprazan could achieve sustained inhibition of gastric acid secretion for up to 24 h (17). Co-administration with certain cardiovascular drugs, such as amlodipine, may affect its metabolic process (18). Voriconazole significantly increases the plasma concentration of vonoprazan, prolongs its elimination half-life, and reduces its clearance (19). Concomitant use with atorvastatin markedly increases the systemic exposure of atorvastatin (20). According to the study by Scarpignato et al., factors such as ethnicity, body weight, and age have minimal influence on vonoprazan exposure, indicating that dose adjustment is generally unnecessary across different populations (21).

**Figure 1:**
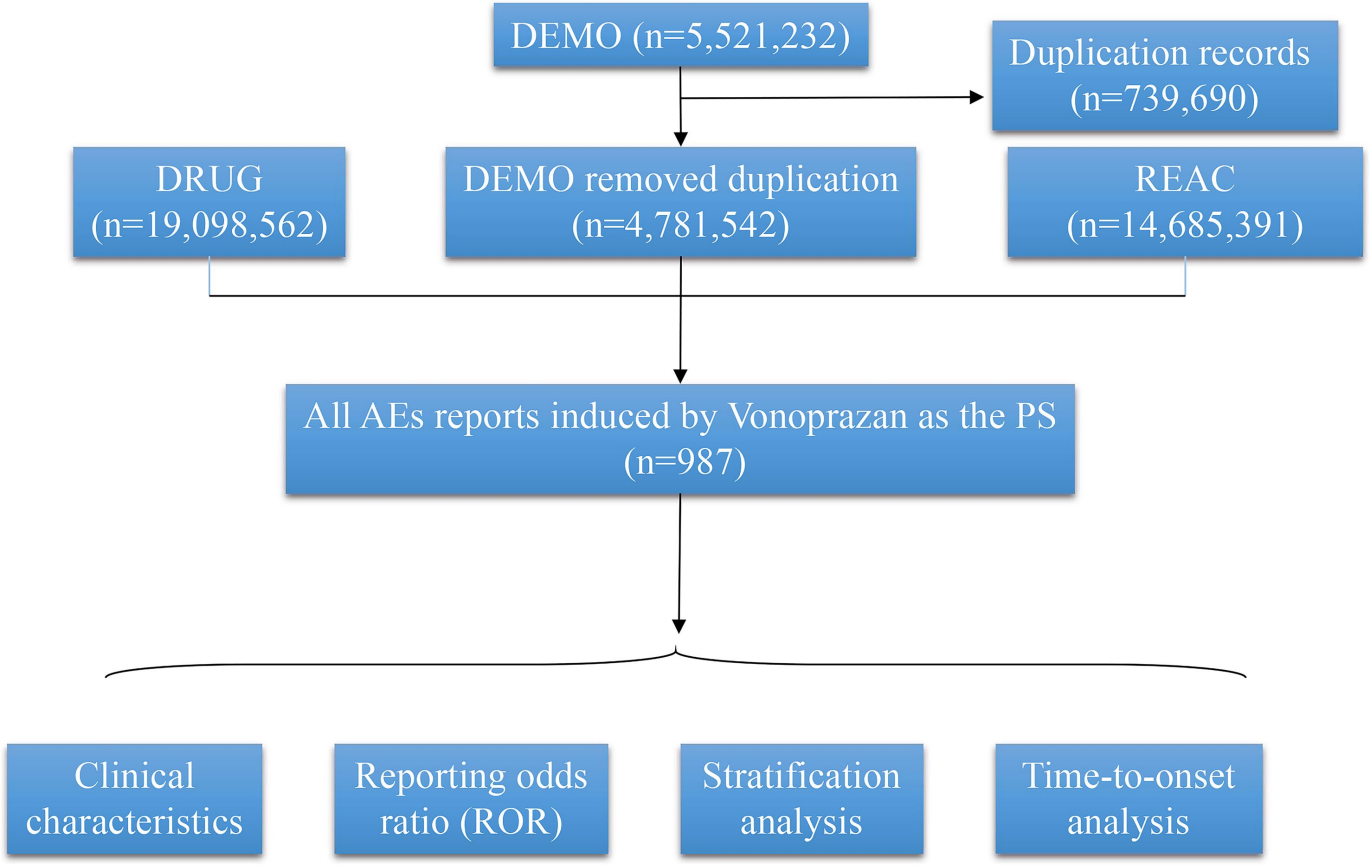
Research flowchart.

Despite its well-established clinical efficacy, the safety profile of vonoprazan warrants continued and systematic evaluation. Early pharmacovigilance efforts have provided preliminary insights but remain limited in scope and representativeness. Ouyang et al. conducted one of the first large-scale pharmacovigilance investigations examining Clostridioides difficile infection (CDI) associated with vonoprazan and proton pump inhibitors (PPIs) using data from both the Japanese Adverse Drug Event Report (JADER) and the U. S. FDA Adverse Event Reporting System (FAERS). Their disproportionality analysis revealed that vonoprazan showed a markedly stronger association with CDI than PPIs, with reporting odds ratios (RORs) of 15.84 (95% CI: 12.23–20.50) in JADER and 11.50 (95% CI: 6.36–20.82) in FAERS. The risk was particularly elevated among elderly patients aged ≥60 years, underscoring the need for targeted monitoring in older populations. However, that study focused exclusively on infectious complications and was constrained by a limited sample size, preventing comprehensive characterization of vonoprazan’s broader safety spectrum. Another study by Chi et al. (22) utilized FAERS data from 2015 to 2022 and identified several high-intensity signals for vonoprazan, such as plateletcrit increased, benign duodenal neoplasm, gallbladder volvulus, and hepatobiliary and metabolic disorders. Although this analysis expanded the range of reported events, it lacked integration of temporal trends, severity stratification, or demographic subgroup analysis, and it predated vonoprazan’s U. S. market approval. Consequently, neither prior study adequately reflects post-approval real-world usage in the United States. Building upon these limitations, the present study employed the FDA Adverse Event Reporting System (FAERS) database to systematically evaluate Vonoprazan. By designating Vonoprazan-associated adverse events reported from the first quarter (Q1) of 2023 through the first quarter (Q1) of 2025- representing the post-approval, real-world clinical phase in the United States. Reports designating vonoprazan as the primary suspected drug were analyzed using four complementary disproportionality algorithms- Reporting Odds Ratio (ROR), Proportional Reporting Ratio (PRR), Bayesian Confidence Propagation Neural Network (BCPNN), and Multi-Item Gamma Poisson Shrinker (MGPS)-to detect potential safety signals. Severity classification, time-to-onset (TTO), and Weibull shape parameter (WSP) modeling were further incorporated to characterize the temporal dynamics of adverse events. Subgroup analyses were conducted by sex and age to identify potential high-risk populations (Figure 1).

Importantly, our analysis identified 16 positive-signal Preferred Terms (PTs)—including facial paralysis, cholecystitis, and oesophageal adenocarcinoma—that are not currently listed in the FDA label for vonoprazan. To our knowledge, this is the first comprehensive, post-approval U. S.-based pharmacovigilance study to (i) compare serious versus non-serious outcomes, (ii) delineate sex- and age-specific risk signals, and (iii) characterize early- versus late-onset adverse events. These findings provide new, hypothesis-generating insights that may inform ongoing safety surveillance, clinical risk assessment, and regulatory evaluation of vonoprazan in routine medical practice.

## 2 Materials and methods

### 2.1 Data downloading

To evaluate adverse events associated with Vonoprazan, we retrieved data from the FDA’s FAERS database,^1^ selecting cases in which “Vonoprazan” was identified as the primary suspect (PS) drug. All reports submitted between the first quarter (Q1) of 2023 through the first quarter (Q1) of 2025 were systematically reviewed, corresponding to the post-approval period following the FDA’s authorization of Vonoprazan.

### 2.2 Data processing

To ensure data accuracy and completeness, we performed systematic cleaning and integration of the seven core data tables in the FAERS database: DEMO, DRUG, REAC, OUTC, RPSR, THER, and INDI. During the standardization, age was converted to years, weight to kilograms, and all drug names were normalized to uppercase letters with special characters removed. Duplicate records were identified using CASEID, PRIMARYID, and the FDA submission date (FDA_DT). When duplicate CASEIDs were present, only the record with the latest FDA_DT was retained; if both CASEID and FDA_DT were identical, the record with the largest PRIMARYID was kept. Deduplication was performed using MySQL (version 8.0.42). Adverse events were coded according to the Medical Dictionary for Regulatory Activities MedDRA (version 24.0) by mapping REAC file entries to standardized Preferred Terms (PTs). Cases missing key demographic variables such as sex and age were flagged and retained for sensitivity analyses. For continuous variables like weight, if the missing rate was <10%, values were imputed using the median from age- and sex-matched subgroups (23). Time-to-onset analysis was conducted as the interval between the drug start date (START_DT) and the adverse event onset date (EVENT_DT). Records with EVENT_DT earlier than START_DT or missing dates were excluded (24). To evaluate temporal patterns of adverse event occurrence, a Weibull distribution model was fitted to onset time data, and the shape parameter (*β*) was used to infer whether risk increased, decreased, or remained constant over time. Comprehensive clinical data, including sex, age, weight, height, reporting country, reporting quarter, and outcome measures, were systematically collected and analyzed. Serious outcomes were defined as death, life-threaten ingevents, hospitalization, disability, or other severe consequences (25). Some cases may exhibited more than one serious outcome, such that the total number of outcomes could exceed the total number of individual cases (26, 27). As FAERS is a spontaneous reporting system, it is inherently subject to underreporting, stimulated reporting, channeling bias (i.e., preferential use of vonoprazan among higher-risk patients), and duplicate or incomplete submissions. To partially mitigate these biases, we (i) implemented a standardized multi-step deduplication protocol using CASEID, FDA_DT, and PRIMARYID; (ii) restricted analyses to cases explicitly listing vonoprazan as the primary suspected drug; and (iii) considered a signal potentially meaningful only if it achieved statistical significance across multiple disproportionality algorithms. Nevertheless, these measures cannot completely eliminate residual confounding by indication or selective reporting.

### 2.3 Statistical methods

The study employed four commonly used disproportionality analysis algorithms -Reported Ratio of Ratios (ROR), Proportionate Reported Ratio of Ratios (PRR), Bayesian Confidence Interval Neural Network Propagation (BCPNN), Multiple Gamma-Poisson Shrinkage estimation (MGPS)-to detect potential drug–event associations. These methods are widely recognized in international pharmacovigilance research and are designed to identify potential safety signals from spontaneous reporting systems. ROR and PRR, representing frequentist approaches, were used for initial signal screening because of their computational simplicity and interpretability, making them suitable for large-scale spontaneous reporting data (28). For ROR, a signal was considered positive if the point estimate was≥ 3 and the lower bound of the 95% confidence interval (CI) exceeded 1. For PRR, positive signals required PRR ≥ 2, *χ*^2^ ≥ 4, and at least three cases for the Preferred Term (PT). Because proportional methods may be affected by collinearity and reporting bias, two Bayesian approaches-BCPNN and MGPS-were additionally applied to enhance the robustness and minimize false-positive detections (29, 30). In the BCPNN method, signals were identified based on the Information Component (IC) and its lower95% credibility limit (IC_025_); with IC_025_ > 0 was considered a positive signal. For the MGPS method, the Empirical Bayes Geometric Mean (EBGM) and its 5th percentile lower bound (EBGM_05_) were used, with EBGM_05_ > 2 and EBGM> 0 indicating a positive signal (24, 27). Differences between serious and non-serious reports were assessed using Pearson chi-square (*χ*^2^) and Fisher’s Exact Test. A two-tailed *p*-value of <0.05 was considered statistical significance. All analyses and data visualizations were performed using R software (version 4.5.0; R Foundation for Statistical Computing, Vienna, Austria). A Preferred Term (PT) was regarded as a “positive signal” if it met predefined criteria in at least one frequentist method (ROR or PRR) and exhibited a directionally consistent association in at least one Bayesian method (BCPNN or MGPS). When a PT fulfilled only one methodological criterion (frequentist or Bayesian), it was retained but interpreted as a hypothesis-generating signal rather than confirmatory evidence. To control for multiple testing, false discovery rate (FDR)–adjusted *p*-values were calculated using the Benjamini–Hochberg procedure. Formal causality assessment frameworks such as the Naranjo algorithm and the WHO-Uppsala Monitoring Center (WHO-UMC) criteria were not applied, as FAERS case narratives frequently lack essential details (e.g., dosing history, dechallenge/rechallenge data, or alternative diagnoses). Therefore, all “signals” identified in this study should be interpreted as disproportional reporting signals rather than established causal relationships.

### 2.4 Time to adverse events, drug comorbidities and Weibull shape parameter analysis

The interval between the initiation of vonoprazan the onset of an adverse events was defined as the time-to-onset (TTO), which was statistically summarized using median and interquartile range (IQR) (31). In parallel, comorbidities and the cumulative incidence of adverse events during the observation period were also evaluated. Because FAERS does not capture comprehensive longitudinal clinical data, formal adjustment for comorbidities or concomitant medications using multivariable regression was not feasible. To qualitatively assess potential confounding, we summarized the most frequently co-administered drugs (e.g., aspirin, anticoagulants, and other acid-suppressing agents) and evaluated whether high-signal Preferred Terms (PTs) plausibly corresponded to the known adverse profiles of these agents—for example, upper gastrointestinal bleeding associated with antiplatelet therapy. This contextual evaluation allowed differentiation between signals likely intrinsic to vonoprazan and those potentially attributable to underlying clinical conditions or co-medications. Subsequently, a Weighted Scoring Procedure (WSP) was applied to model temporal trends in adverse event occurrence. The estimated *α*- and *β*-parameters were used to infer event risk patterns over time, where *β* < 1 indicates early failure (decreasing risk), *β* = 1 suggests a constant risk, and *β* > 1 reflects a late-onset pattern (32, 33).

## 3 Results

### 3.1 Descriptive analysis of data

**Figure 2:**
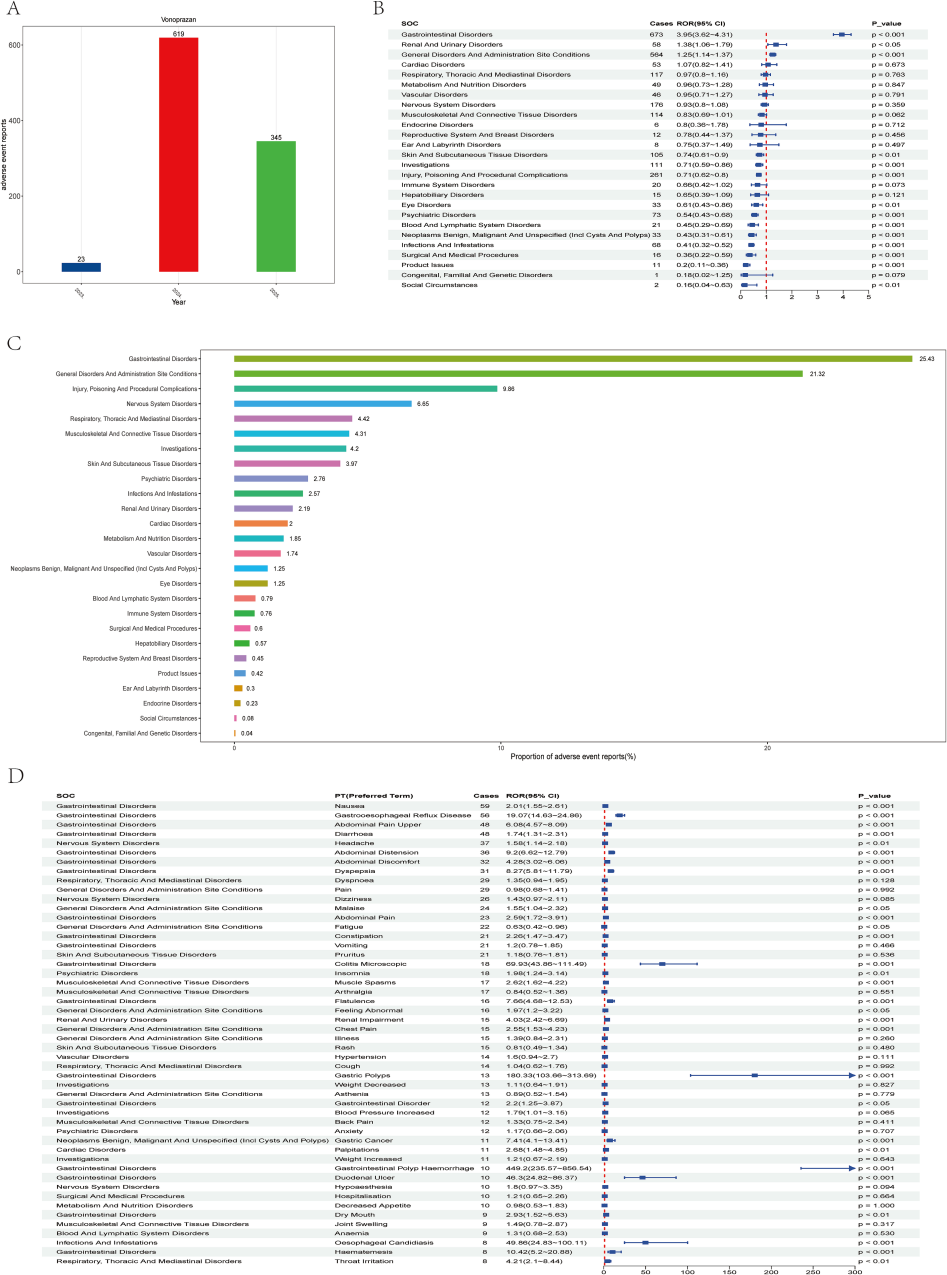
Analysis of vonoprazan-related adverse events using the faers database. **(A)** Annual trends in adverse event report frequency from 2023 to 2025. **(B)** Safety signal evaluation of vonoprazan-associated systemic adverse events by system organ class (SOC), ranked by reporting odds ratio (ROR) and 95% CI. **(C)** Proportional distribution of reports across different soc categories. **(D)** Distribution of adverse events by Preferred Term (PT), organized by the number of reported cases.

Between the first quarter (Q1) of 2023 and the first quarter (Q1) of 2025, a total of 5,521,232 adverse event (AE) reports were retrieved from the FAERS database. After excluding duplicate entries, 4,781,542 unique reports were retained. Among these, 987 reports identified Vonoprazan as the primary suspected drug (Figure 1). Of these, 292 (29.6%) reports involved male patients and 612 (62.0%) involved female patients. Regarding patient demographics, 160 individuals (16.2%) weighed between 50 and 100 kg, 361 (36.6%) were aged 18–64.9 years, and 250 (25.3%) were aged 65–85 years. The majority of reports were submitted by consumers (64.8%), followed by physician (19.4%). Serious adverse events (SAEs), including hospitalization, fatality, and disability, were documented in 338 cases, among which hospitalizations occurred in 85 (25.1%). Most reports originated from the United States (675, 68.4%), with 619 events (62.7%) reported in 2024 alone (Table 1; Figure 2A).

**Table 1:** Baseline information of serious and non-serious adverse events.

Variable	NO_Serious	Serious	Overall
	(*N* = 649)	(*N* = 338)	(*N* = 987)
Gender			
Female	449 (69.2%)	163 (48.2%)	612 (62.0%)
Male	160 (24.7%)	132 (39.1%)	292 (29.6%)
Missing	40 (6.2%)	43 (12.7%)	83 (8.4%)
Age			
<18 years	5 (0.8%)	75 (22.2%)	80 (8.1%)
≥18 and < 64.9 years	306 (47.1%)	55 (16.3%)	361 (36.6%)
≥65 and < 85 years	173 (26.7%)	77 (22.8%)	250 (25.3%)
≥85 years	7 (1.1%)	21 (6.2%)	28 (2.8%)
Missing	158 (24.3%)	110 (32.5%)	268 (27.2%)
Weight			
<50 kg	4 (0.6%)	12 (3.6%)	16 (1.6%)
≥50 and <100 kg	93 (14.3%)	67 (19.8%)	160 (16.2%)
≥100 kg	11 (1.7%)	2 (0.6%)	13 (1.3%)
Missing	541 (83.4%)	257 (76.0%)	798 (80.9%)
Outcome			
Other	649 (100%)	226 (66.9%)	875 (88.7%)
Death	0 (0%)	15 (4.4%)	15 (1.5%)
Disability	0 (0%)	4 (1.2%)	4 (0.4%)
Hospitalization	0 (0%)	85 (25.1%)	85 (8.6%)
Life-Threatening	0 (0%)	8 (2.4%)	8 (0.8%)
Reporter_Country			
United States of America	645 (99.4%)	30 (8.9%)	675 (68.4%)
Japan	3 (0.5%)	237 (70.1%)	240 (24.3%)
Brazil	1 (0.2%)	56 (16.6%)	57 (5.8%)
China	0 (0%)	14 (4.1%)	14 (1.4%)
Fatal_or_NO_Fatal			
No	649 (100%)	323 (95.6%)	972 (98.5%)
Yes	0 (0%)	15 (4.4%)	15 (1.5%)
Reportertype			
Consumer	551 (84.9%)	89 (26.3%)	640 (64.8%)
Physician	44 (6.8%)	147 (43.5%)	191 (19.4%)
Health Professional	45 (6.9%)	51 (15.1%)	96 (9.7%)
Pharmacist	6 (0.9%)	50 (14.8%)	56 (5.7%)
Missing	3 (0.5%)	1 (0.3%)	4 (0.4%)
GetDataYear			
2023	0 (0%)	23 (6.8%)	23 (2.3%)
2024	371 (57.2%)	248 (73.4%)	619 (62.7%)
2025	278 (42.8%)	67 (19.8%)	345 (35.0%)

### 3.2 Adverse event signal detection and interpretation

In the system organ class (SOC)**-**, level analysis a total of 26 SOC categories were identified as being affected. Among these, *Gastrointestinal Disorders* was the most frequently reported SOC, accounting for 25.43% of all cases (Figures 2B,C). This SOC exhibited positive disproportionality signals across all four detection algorithms. A chi-square (*χ*^2^) test was applied to evaluate distributional differences across the14 major SOC. SOCs with more than 100 cases included *Gastrointestinal Disorders, General Disorders and Administration Site Conditions, Injury, Poisoning and Procedural Complications, Nervous System Disorders, Respiratory, Thoracic and Mediastinal Disorders, Musculoskeletal and Connective Tissue Disorders, Investigations*, and *Skin and Subcutaneous Tissue Disorders*.

The study first examined the top 50 most frequently reported adverse events, ranked in descending order by the number of Preferred Term (PT) cases, and identified 29 PTs with statistically significant associations (*p* < 0.05). Among these, he most common events (>30 cases) were Nausea (ROR = 2.01, 95% CI: 1.55–2.61), Gastroesophageal Reflux Disease (GERD; ROR 19.07, 95% CI: 14.63–24.86), Abdominal Pain Upper (ROR 6.08, 95% CI: 4.57–8.09), diarrhea (ROR 1.74, 95% CI: 1.31–2.31), Headache (ROR 1.58, 95% CI: 1.14–2.18), Abdominal Distension (ROR 9.20, 95% CI: 6.62–12.79), Abdominal Discomfort (ROR 4.28, 95% CI: 3.02–6.06), and Dyspepsia (ROR 8.27, 95% CI: 5.81–11.79; Figure 2D).

**Figure 3:**
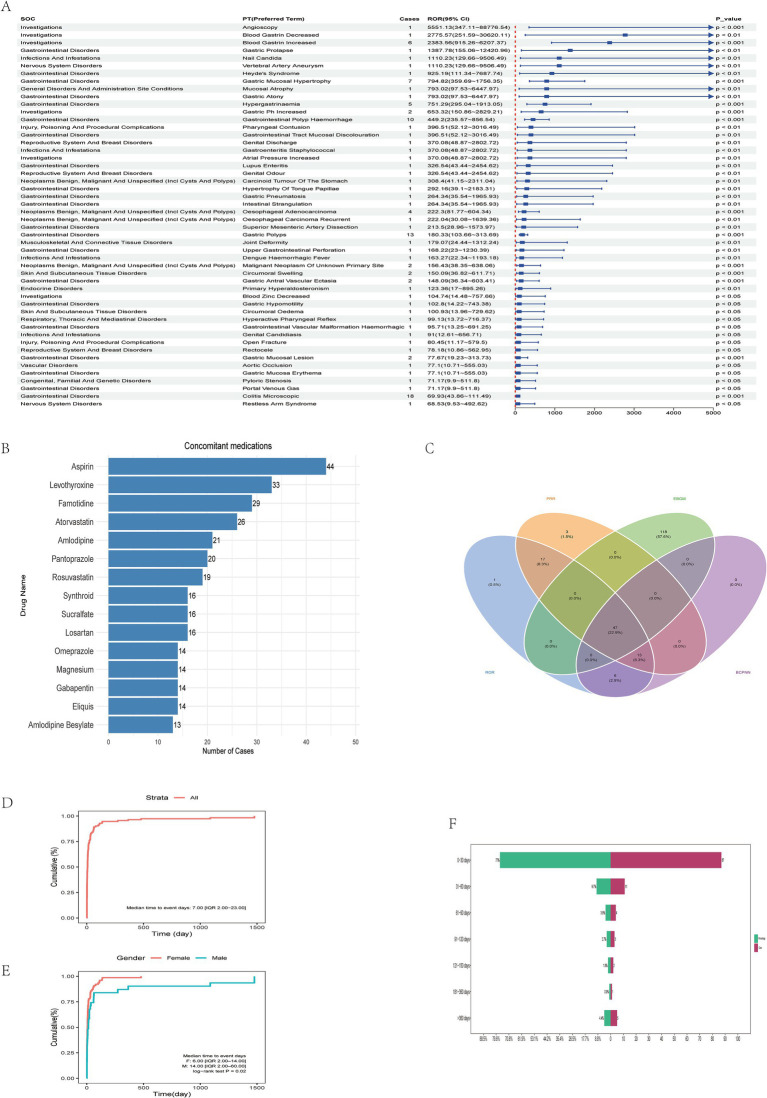
Multi-Method detection and characterization of vonoprazan-related adverse reactions. **(A)** Ranking of Preferred Terms (PTs) according to ROR values. **(B)** Analysis of commonly co-administered drugs with vonoprazan. **(C)** Cross-validation of vonoprazan-related adverse events using four disproportionality algorithms: Reporting Odds Ratio (ROR), Proportional Reporting Ratio (PRR), Bayesian Lower Bound (EBGM), and Bayesian Confidence Propagation Neural Network (BCPNN). **(D)** Cumulative incidence curve showing a median time-to-onset (TTO) of 7 days (IQR: 2.00–23.00). **(E)** Gender-stratified cumulative incidence curve showing a median TTO of 6 days (IQR: 2.00–14.00) in females and 14 days (IQR: 2.00–60.00) in males (log-rank test, *p* = 0.02). **(F)** Analysis of the time to onset of adverse drug events associated with Vonoprazan.

Subsequently, the top 50 PTs with the highest reporting odds ratios (RORs) were ranked in descending order, all exhibiting *p* < 0.05 and ROR > 68.53. Among these, PTs reported in ≥5 cases included *Blood Gastrin Increased* (ROR 2383.56, 95% CI: 915.26–6207.37; Figure 3A), *Gastric Mucosal Hypertrophy* (ROR 794.82, 95% CI: 359.69–1756.35), *Hypergastrinemia* (ROR 751.29, 95% CI: 295.04–1913.05), *Gastrointestinal Polyp Hemorrhage* (ROR 449.2, 95% CI: 235.57–856.54), and *Gastric Polyps* (ROR 180.33, 95% CI: 103.66–313.69), as well as *Colitis Microscopic* (ROR 69.93, 95% CI: 43.86–111.49; Most vonoprazan-related adverse reactions are non-serious and gastrointestinal, but rare severe events—especially renal impairment and Abdominal pain upper—occur, warranting closer monitoring in high-risk or concomitantly treated patients. Table 2).

**Table 2:** Comparison of serious and non-serious events.

Variable	Serious	NO_Serious	*p*_value	Statistic	Test
Cases	338	649			
PT	1,137	1,509			
Abdominal distension	4 (0.4%)	32 (2.1%)	*p* < 0.001	13.827	Chi-Square
Abdominal pain upper	13 (1.1%)	35 (2.3%)	*p* < 0.05	4.397	Chi-Square
Asthenia	1 (0.1%)	12 (0.8%)	*p* < 0.05	5.267	Chi-Square
Blood pressure decreased	7 (0.6%)	1 (0.1%)	*p* < 0.05	–	Fisher’s Exact Test
Dizziness	5 (0.4%)	21 (1.4%)	*p* < 0.05	5.100	Chi-Square
Dyspepsia	5 (0.4%)	26 (1.7%)	*p* < 0.01	8.147	Chi-Square
Fatigue	2 (0.2%)	20 (1.3%)	*p* < 0.01	9.044	Chi-Square
Flatulence	1 (0.1%)	15 (1.0%)	*p* < 0.01	7.414	Chi-Square
Gastrooesophageal reflux disease	11 (1.0%)	45 (3.0%)	*p* < 0.001	11.750	Chi-Square
Haematemesis	7 (0.6%)	1 (0.1%)	*p* < 0.05	–	Fisher’s Exact Test
Headache	4 (0.4%)	33 (2.2%)	*p* < 0.001	14.534	Chi-Square
Illness	2 (0.2%)	13 (0.9%)	*p* < 0.05	4.259	Chi-Square
Nausea	11 (1.0%)	48 (3.2%)	*p* < 0.001	13.575	Chi-Square
Palpitations	1 (0.1%)	10 (0.7%)	*p* < 0.05	–	Fisher’s Exact Test
Rash	2 (0.2%)	13 (0.9%)	*p* < 0.05	4.259	Chi-Square
Renal impairment	13 (1.1%)	2 (0.1%)	*p* < 0.01	10.029	Chi-Square
Weight increased	1 (0.1%)	10 (0.7%)	*p* < 0.05	–	Fisher’s Exact Test

Cross-validation using all four disproportionality algorithms (ROR, PRR, BCPNN, and MGPS) identified 47 PTs meeting the significance criterion (*p* < 0.05; Figure 3C). Comparison with the official FDA drug label^2^ revealed 16 previously unlisted adverse events spanning multiple organ systems. These newly identified events include Var*icose Vein, Throat Irritation, Throat Tightness, Dry Throat, Near Death Experience, Dementia, Facial Paralysis, Oesophageal Adenocarcinoma, Neuroendocrine Tumor, Musculoskeletal Discomfort, Sjogren’s Syndrome, Blood Magnesium Decreased, Cholecystitis, Polyp, Malabsorption*, and *Hiatus Hernia*. These novel PTs may represent potential, previously unrecognized safety risks associated with vonoprazan. Symptoms such as *Throat Irritation, Throat Tightness*, and *Dry Throat* may reflect mucosal or immune-mediated responses triggered by local irritation or systemic inflammation. Events such as *Dementia* and *Facial Paralysis* may indicate involvement of the central or peripheral nervous system, warranting further evaluation for potential neurotoxicity. The occurrence of *Oesophageal Adenocarcinoma* and *Neuroendocrine tumor* suggests possible carcinogenic potential, although causality cannot be inferred from spontaneous reporting data. Additionally, *Sjogren’s Syndrome*, *Cholecystitis*, and *Malabsorption* may be related to autoimmune or gastrointestinal mechanisms. Although these PTs are not currently included in regulatory safety documentation, their repeated occurrence in FAERS supports the need for heightened pharmacovigilance. Furthermore, three PTs remained significant after false discovery rate (FDR) correction: oesophageal adenocarcinoma (ROR = 222.3; 95% CI: 81.77–604.34), neuroendocrine tumor (ROR = 45.04; 95% CI: 14.45–140.39), and polyp (ROR = 18.59; 95% CI: 7.72–44.77) (Table 3). Although these PTs exhibited large effect estimates, the wide confidence intervals reflected the small number of cases. These statistically unstable yet clinically relevant signals should therefore be interpreted as hypothesis-generating rather than confirmatory.

**Table 3:** Positive signals identified by four algorithms.

**soc_name**	**PT**	**Cases**	**ROR(95%Cl)**	**PRR(χ** ^**2**^ **)**	**EBGM(EBGM05)**	**IC(IC025)**	**Bonferroni-adjusted P value**
Vascular Disorders	Varicose vein	3	15.5(4.99-48.17)	15.49(40.54)	15.45(4.97)	3.95(0.3)	0.71
Skin And Subcutaneous Tissue Disorders	Stevens-Johnson Syndrome	5	8.74(3.63-21.04)	8.73(34.16)	8.72(3.62)	3.12(0.75)	0.22
Respiratory, Thoracic And Mediastinal Disorders	Throat irritation	8	4.21(2.1-8.44)	4.2(19.54)	4.2(2.1)	2.07(0.67)	0.54
	Throat tightness	5	5.25(2.18-12.63)	5.24(17.16)	5.24(2.18)	2.39(0.44)	1.00
	Dry throat	3	7.52(2.42-23.35)	7.51(16.92)	7.5(2.42)	2.91(0.07)	1.00
Renal And Urinary Disorders	Renal impairment	15	4.03(2.42-6.69)	4.01(33.89)	4.01(2.41)	2(1.03)	***p<0.01***
Psychiatric Disorders	Near death experience	3	11.18(3.6-34.72)	11.17(27.71)	11.14(3.59)	3.48(0.21)	1.00
Nervous System Disorders	Dementia	6	4.62(2.07-10.29)	4.61(16.96)	4.61(2.07)	2.2(0.51)	1.00
	Facial paralysis	3	7.59(2.44-23.57)	7.58(17.13)	7.58(2.44)	2.92(0.07)	1.00
Neoplasms Benign, Malignant And Unspecified (Incl Cysts And Polyps)	Gastric cancer	11	7.41(4.1-13.41)	7.39(60.69)	7.38(4.08)	2.88(1.43)	***p<0.001***
	Oesophageal adenocarcinoma	4	222.3(81.77-604.34)	221.96(846.03)	213.46(78.52)	7.74(0.97)	***p<0.001***
	Neuroendocrine tumor	3	45.04(14.45-140.39)	44.99(128.01)	44.64(14.32)	5.48(0.45)	***p<0.05***
Musculoskeletal And Connective Tissue Disorders	Musculoskeletal discomfort	4	5.37(2.01-14.32)	5.36(14.18)	5.36(2.01)	2.42(0.22)	1.00
	Sjogren’s syndrome	3	12.41(3.99-38.55)	12.4(31.36)	12.37(3.98)	3.63(0.24)	1.00
Metabolism And Nutrition Disorders	Hypomagnesaemia	6	9.23(4.14-20.57)	9.21(43.83)	9.19(4.12)	3.2(0.99)	***p<0.05***
Investigations	Blood gastrin increased	6	2383.56(915.26-6207.37)	2378.16(9979.88)	1665.01(639.34)	10.7(1.54)	***p<0.001***
	Blood magnesium decreased	3	7.58(2.44-23.53)	7.57(17.09)	7.56(2.44)	2.92(0.07)	1.00
Infections And Infestations	Oesophageal Candidiasis	8	49.86(24.83-100.11)	49.71(378.48)	49.28(24.54)	5.62(1.98)	***p<0.001***
Hepatobiliary Disorders	Cholecystitis	3	8.07(2.6-25.06)	8.06(18.53)	8.05(2.59)	3.01(0.1)	1.00
General Disorders And Administration Site Conditions	Thirst	7	8.49(4.04-17.84)	8.47(46.06)	8.46(4.03)	3.08(1.11)	***p<0.05***
	Polyp	5	18.59(7.72-44.77)	18.56(82.79)	18.5(7.68)	4.21(1.06)	***p<0.01***
Gastrointestinal Disorders	Gastrooesophageal reflux disease	56	19.07(14.63-24.86)	18.68(935.2)	18.62(14.29)	4.22(3.44)	***p<0.001***
	Abdominal pain upper	48	6.08(4.57-8.09)	5.99(199.98)	5.99(4.5)	2.58(2.03)	***p<0.001***
	Abdominal distension	36	9.2(6.62-12.79)	9.09(259.23)	9.08(6.53)	3.18(2.42)	***p<0.001***
	Abdominal discomfort	32	4.28(3.02-6.06)	4.24(79.38)	4.24(2.99)	2.08(1.44)	***p<0.001***
	Dyspepsia	31	8.27(5.81-11.79)	8.19(195.64)	8.18(5.74)	3.03(2.23)	***p<0.001***
	Colitis microscopic	18	69.93(43.86-111.49)	69.46(1199.63)	68.61(43.04)	6.1(3.24)	***p<0.001***
	Flatulence	16	7.66(4.68-12.53)	7.62(91.97)	7.61(4.65)	2.93(1.75)	***p<0.001***
	Gastric polyps	13	180.33(103.66-313.69)	179.45(2234.68)	173.86(99.94)	7.44(2.92)	***p<0.001***
	Gastrointestinal polyp hemorrhage	10	449.2(235.57-856.54)	447.5(4122.62)	414.18(217.21)	8.69(2.52)	***p<0.001***
	Duodenal ulcer	10	46.3(24.82-86.37)	46.13(437.88)	45.75(24.53)	5.52(2.3)	***p<0.001***
	Haematemesis	8	10.42(5.2-20.88)	10.39(67.81)	10.38(5.18)	3.38(1.38)	***p<0.01***
	Gastric mucosal hypertrophy	7	794.82(359.69-1756.35)	792.72(4843.18)	693.75(313.95)	9.44(1.89)	***p<0.001***
	Feces discolored	7	9.81(4.67-20.6)	9.78(55.11)	9.77(4.65)	3.29(1.2)	***p<0.01***
	Eructation	6	7.96(3.57-17.74)	7.94(36.37)	7.93(3.56)	2.99(0.9)	0.09
	Hypergastrinaemia	5	751.29(295.04-1913.05)	749.87(3294.2)	660.72(259.48)	9.37(1.32)	***p<0.001***
	Oesophagitis	5	14.3(5.94-34.44)	14.28(61.6)	14.25(5.92)	3.83(0.97)	***p<0.05***
	gastrointestinal pain	5	10.48(4.36-25.23)	10.47(42.73)	10.45(4.34)	3.39(0.84)	0.09
	Gastritis	5	5.62(2.34-13.52)	5.61(18.93)	5.6(2.33)	2.49(0.48)	1.00
	Oesophageal disorder	4	35.34(13.21-94.52)	35.29(132.43)	35.07(13.11)	5.13(0.87)	***p<0.01***
	Oesophageal pain	4	30.62(11.45-81.86)	30.57(113.8)	30.41(11.37)	4.93(0.85)	***p<0.01***
	Gastric hemorrhage	4	13.86(5.19-37)	13.84(47.53)	13.81(5.17)	3.79(0.66)	0.16
	Gastric ulcer	4	6.99(2.62-18.65)	6.98(20.48)	6.97(2.61)	2.8(0.37)	1.00
	Paraesthesia oral	4	6.98(2.62-18.62)	6.97(20.44)	6.96(2.61)	2.8(0.37)	1.00
	Gastric ulcer hemorrhage	3	26.54(8.53-82.56)	26.51(73.29)	26.39(8.48)	4.72(0.4)	0.15
	Malabsorption	3	16.48(5.3-51.23)	16.47(43.45)	16.42(5.28)	4.04(0.31)	0.60
	Hiatus hernia	3	8.09(2.6-25.11)	8.08(18.58)	8.07(2.6)	3.01(0.1)	1.00

### 3.3 Stratified analysis

**Figure 4:**
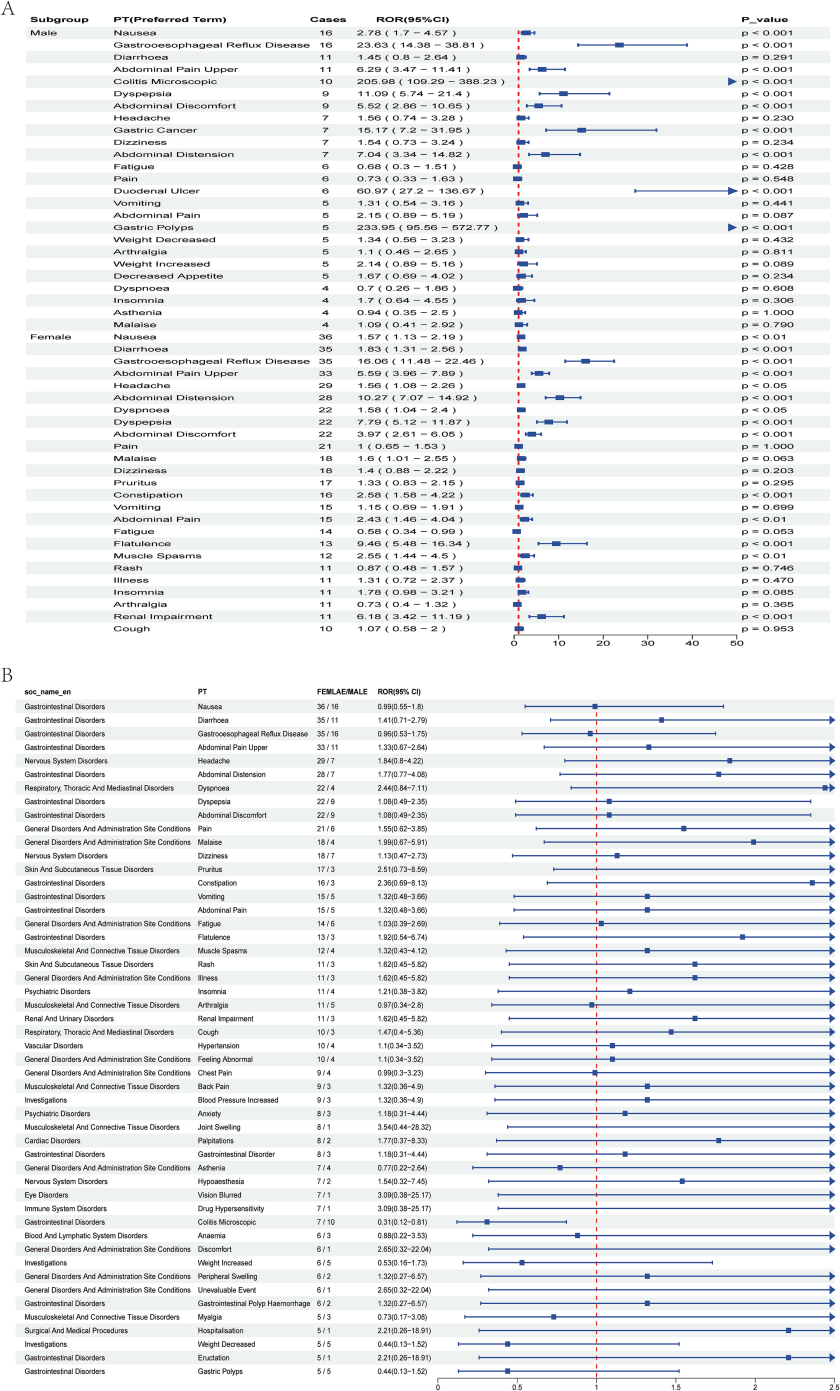
Signal analysis of Vonoprazan-related adverse events by gender. **(A)** Top 25 adverse events in male and female subgroups. **(B)** Top 50 adverse events stratified by gender, compared by Reporting Odds Ratio (ROR) and 95% CI.

**Figure 5:**
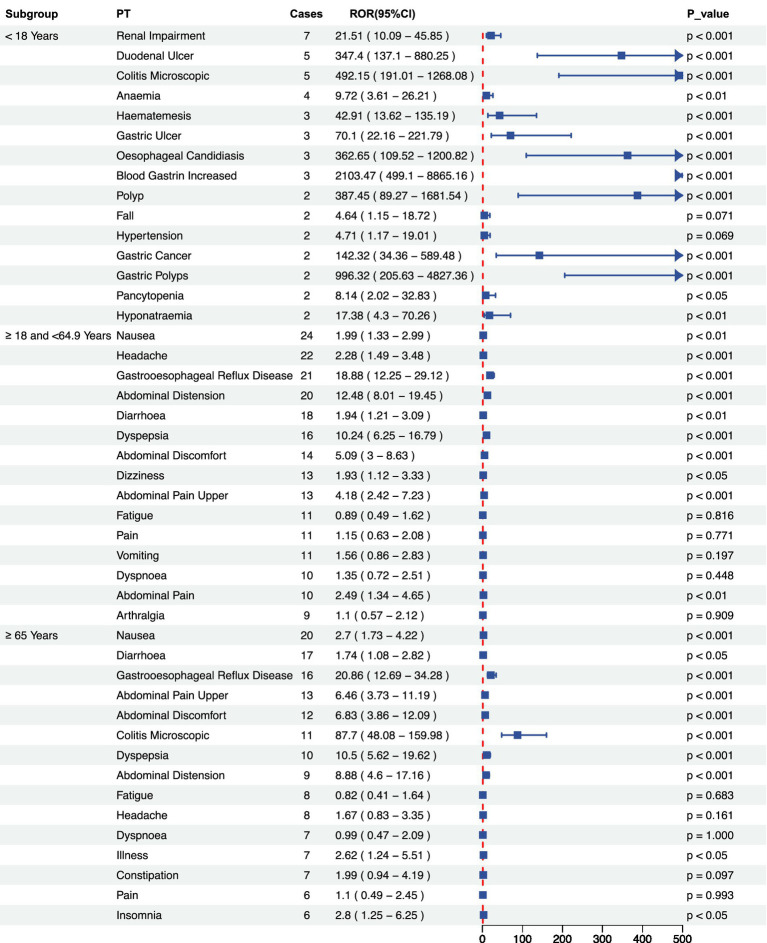
Signal intensity analysis of Vonoprazan-related adverse across age subgroups. Reporting Odds Ratio (ROR) and 95% confidence level. ROR and their 95% confidence intervals for adverse events (Preferred Terms, PTs) in three age subgroups: <18 years, 18–64.9 years, and ≥65 years.

Stratified analysis by gender revealed that the overall incidence of adverse events was significantly higher among females than males. In the male subgroup several high-risk signals were observed-particularly for *gastric polyps*, *colitis microscopic*, and *gastric cancer*- indicating possible severe gastrointestinal pathology that warrants close clinical attention. Conversely, the female subgroup predominantly exhibited typical gastrointestinal symptoms, including *gastroesophageal reflux disease* (GERD), *abdominal distension*, and *dyspepsia* (Figures 4A,B). However, a significant association with *renal impairment* was also detected, underscoring the need for further longitudinal monitoring. Age-stratified analysis revealed several severe events with extremely high Reporting Odds Ratios (ROR) among patients <18 years, most notably *blood gastrin increased* (ROR 2103.47, 95% CI: 499.1–8865.16). In the 18- 64 years subgroup, most adverse events were common gastrointestinal disorders with statistically significant positive associations. Among patients aged ≥65 years, adverse events were significant correlation with serious gastrointestinal outcomes, particularly inflammatory and reflux-related conditions, suggesting that dose reduction or gradual tapering strategies maybe advisable (Figure 5).

### 3.4 Duration of adverse events, co-administration and WSP analysis

The median duration of adverse events was 7 days (interquartile range: 2–23 days), consistent with the time-to-onset (TTO) analysis, which indicated that the most events occurred within 30 days of drug initiation (Figure 3F; Table 4). The most frequently co-administered drugs were *Aspirin, Levothyroxine*, and *Famotidine*, with acid-suppressing and anticoagulant agents being the most prevalent (Figure 3B). Cumulative incidence analysis demonstrated that adverse events predominantly occurred early during treatment, with females experiencing them significantly earlier than males (Figures 3D,E). The Weibull survival probability (WSP) analysis confirmed an “early failure” pattern, indicated that the hazard of adverse reactions declined progressively over time (Table 4).

**Table 4:** Weibull distribution.

Cases	TTO	Weibull distribution		Failure type
	Median (IQR)	Scale parameter	Shape parameter	
		α (95% CI)	β (95% CI)	
113	7 (2–23)	19.73 (11.79–27.67)	0.49 (0.43–0.55)	Early failure

## 4 Discussion

This study employed data from the U. S. Food and Drug Administration’s Adverse Event Reporting System (FAERS) to perform a comprehensive pharmacovigilance assessment of Vonoprazan, encompassing all adverse events (AE) reported submitted between the first quarter (Q1) of 2023 and the first quarter (Q1) of 2025. The findings demonstrated that Vonoprazan was associated with a broad spectrum of adverse events in real-world clinical practice, with gastrointestinal system disorders being the most frequently reported. Common AEs include *nausea, epigastric pain, and abdominal discomfort*, each reported in more than 30 cases with reporting odds ratios (RORs) exceeding 1.5 (Figure 2D). A substantial proportion of these events were classified as serious, underscoring the need for strengthened pharmacovigilance and clinical monitoring during vonoprazan therapy. Compared with previous FAERS-based analyses of vonoprazan that primarily focused on infection-related outcomes during its early post-marketing phase and were constrained by smaller sample sizes (12), the present study provides an updated and more granular evaluation of its post-approval safety profile in the United States. Specifically, by analyzing all cases in which vonoprazan was designated as the primary suspected drug from Q1 2023 to Q1 2025, we were able to (i) differentiate serious from non-serious outcomes, (ii) identify sex- and age-specific high-risk signals, (iii) characterize temporal onset patterns using time-to-onset and Weibull modeling, and (iv) propose 16 candidate safety signals not yet incorporated into FDA labeling. These methodological features extend beyond traditional disproportionality screening and aim to inform evidence-based decision-making by both prescribers and regulatory authorities.

In the demographic analysis, the proportion of female patients reporting vonoprazan-related AEs was significantly higher than that of males, a trend consistent with the higher prevalence of *gastroesophageal reflux disease* (GERD) among females. This phenomenon may be partly attributable to hormonal fluctuations influencing lower esophageal sphincter tone in women (34, 35). The majority of vonoprazan users were aged 18–65 years; however, t patients aged ≥65 years accounted for more than 25% of the cohort. Older adults exhibited a higher incidence of AEs related to inflammatory or structural gastrointestinal disorders, including *gastric polyps, microscopic colitis,* and *gastric cancer* (36, 37). For this population, clinicians should carefully evaluate comorbidities and concomitant medications. In cases requiring long-term therapy, periodic endoscopic surveillance and reassessment of treatment necessity may be appropriate in selected patients. Minimizing treatment duration and maintaining the lowest effective dose may further mitigate the potential risk of mucosal hypertrophy or neoplastic transformation.

Through a system organ classification (SOC) analysis, gastrointestinal system were identified as the most frequently reported adverse reactions associated with vonoprazan (25.43%), a finding intrinsically related to its pharmacological mechanism. As a potassium-competitive acid blocker (P-CAB), Vonoprazan exerts rapid and potent inhibition of gastric acid secretion. However, such potent acid suppression can trigger compensatory hypergastrinemia, which may contribute to gastric mucosal hypertrophy, gastric polyp formation, gastrointestinal hemorrhage, and gastrointestinal malignancies. The elevated reporting odds ratios (RORs) observed for “*elevated blood gastrin*,” “*gastric mucosal hypertrophy*,” and “*gastric polyp bleeding*” in this study provide pharmacovigilance support for this biological mechanism. Comparable adverse effects have been reported among patients receiving long-term proton pump inhibitor (PPI) therapy. Given Vonoprazan’s stronger and more sustained acid-suppressive potency compared with PPIs, heightened clinical vigilance regarding its potential long-term risks is warranted. Mechanistically, the potent and sustained acid suppression induced by vonoprazan may elicit compensatory hypergastrinemia, resulting in mucosal hypertrophy and polyp formation, which can predispose patients to upper gastrointestinal bleeding and, potentially, neoplastic transformation. This mechanism aligns with biological observations from long-term proton pump inhibitor (PPI) exposure, in which chronic hypoacidity has been associated with hyperplasia of enterochromaffin-like cells, microscopic colitis, and impaired absorption of essential micronutrients. Similar pathophysiological mechanisms may underlie the high reporting odds ratio (ROR) signals observed for blood gastrin increased, gastric mucosal hypertrophy, gastrointestinal polyp hemorrhage, and gastric polyps.

From a clinical perspective, several pragmatic considerations emerge from these signal-level observations. These should be regarded as hypothesis-generating rather than prescriptive recommendations: (1) Use the lowest effective dose and avoid prolonged empiric therapy without periodic reassessment. (2) Schedule early follow-up (within 1–2 weeks of initiation) to detect intolerance or early adverse reactions, consistent with the observed “early-failure” onset pattern. (3) In elderly or long-term users, consider periodic endoscopic surveillance or symptom-driven escalation to endoscopy, particularly in the presence of alarm symptoms such as bleeding, dysphagia, or weight loss. (4) Assess concurrent antiplatelet or anticoagulant therapy (e.g., aspirin, warfarin) and evaluate bleeding risk before initiating vonoprazan. (5) Monitor biochemical markers, such as fasting serum gastrin, in adolescents or long-term users to identify excessive trophic stimulation of the gastric mucosa.

Bunchorntavakul et al. (38) demonstrated that Vonoprazan achieved a markedly higher *Helicobacter pylori* eradication rate compared withproton pump inhibitors (PPIs), with an overall clearance rate of 96.7%. Reported adverse events associated with Vonoprazan included a bitter taste, nausea, abdominal distension, diarrhea, abdominal pain, and dizziness. Similarly, Ang et al. (39) reported comparable adverse events, consistent with the findings of Bunchorntavakul et al. Serious adverse events (SAEs) occurred in fewer than 2% of patients and, included hematological disorders, bone fractures, infections, and prolonged cardiac QT interval. Furthermore, our analysis identified 16 potential *de novo* adverse events not previously documented in the FDA -approved labeling, including *facial paralysis*, *desiccation syndrome, esophageal adenocarcinoma, neuroendocrine tumors*, and *malabsorption syndrome*. Among these, *esophageal adenocarcinoma* and *neuroendocrine tumors* demonstrated statistically significant associations after false discovery rate correction. Although causality cannot be definitively established due to the limited sample size, these potentially serious outcomes among long-term vonoprazan users warrant heightened clinical vigilance and prospective evaluation. Previous research has shown that prolonged acid suppression may impair the absorption of vitamins and minerals, thereby disrupting immune homeostasis and epithelial regeneration within the gastrointestinal mucosa. Such disruption may promote intestinal dysbiosis, aberrant epithelial proliferation, and, in rare cases, tumorigenesis (40). Events such as dementia, facial paralysis, and cholecystitis may instead reflect underlying neurological, vascular, or biliary comorbidities, or potential drug–drug interactions, rather than a direct causal effect of vonoprazan. These should therefore be interpreted as pharmacovigilance signals awaiting clinical validation.

Stratified analyses revealed that male patients reported fewer adverse events overall compared with females. However, high Reporting Odds Ratio (ROR) signals among males were predominantly associated with organic pathologies, including *gastric cancer*, *colitis*, and *gastric polyps*, indicating a potentially higher vonoprazan-related risk in this subgroup. Conversely, female patients were more likely to experience functional gastrointestinal symptoms, such as *nausea*, *bloating*, and *reflux*. Among adolescents (<18 years), adverse events were less frequent overall; however, notably high ROR signal were detected for biochemical abnormalities such as *blood gastrin* increased. In pediatric and adolescent populations, careful consideration of dose selection and treatment duration is warranted. Periodic monitoring of biochemical parameters, such as serum gastrin levels, may be advisable during prolonged therapy. In the elderly patients, vonoprazan use was significantly associated with inflammatory and reflux -related disorders, underscoring the importance of vigilant monitoring and individualized risk assessment in this population.

The comparative analysis of serious versus non-serious cases revealed that Vonoprazan-associated serious adverse events (SAEs), including *hypotension, hematemesis,* and *renal impairment*, although uncommon, were linked to unfavorable prognoses and warrant clinical prioritization. Conversely, mild AEs such as *nausea*, *epigastric pain*, *reflux*, and *headache* were more frequently reported among non-serious cases. This findings suggests that vonoprazan is generally well tolerated but requires careful evaluation in high-risk populations.

Temporal analysis of adverse drug reactions (ADRs) revealed a median onset time of 7 days, with clustering within the first 30 days. Accordingly, clinicians should schedule follow-up visits within 1–2 weeks of therapy initiation to identify early intolerance or serious reactions. Educating Patients on early warning symptoms and integrating digital health tools (e.g., medication diaries or alert systems) may facilitate timely recognition and management of emerging adverse reactions. This pattern corresponds to the “early failure” model (Weibull shape parameter *β*<1), indicating that the initial phase of treatment represents the highest-risk period. This pattern is consistent with the behavior of immunosuppressive agents such as Canakinumab, as documented in the FAERS database, underscoring the necessity for enhanced clinical monitoring during the initial dosing phase (41). Furthermore, the analysis of concomitant drug administration revealed that aspirin, levothyroxine, and famotidine were frequently co-prescribed, given the frequent co-administration of antiplatelet or anticoagulant agents such as aspirin, clinicians should proactively assess gastrointestinal bleeding risk before initiating Vonoprazan. Co-prescription of gastroprotective agents or periodic fecal occult blood testing may be considered in high-risk patients. A multidisciplinary strategy involving cardiology and gastroenterology specialists may optimize therapeutic efficacy while mitigating adverse outcomes. These patterns indicate that part of the observed signal for severe gastrointestinal outcomes—such as haematemesis—may reflect underlying cardiovascular comorbidities and concomitant antiplatelet or anticoagulant use rather than vonoprazan monotherapy.

Identification of 16 Preferred Terms (PTs) absent from current U. S. prescribing information—including facial paralysis, oesophageal adenocarcinoma, neuroendocrine tumor, Sjögren’s syndrome, and malabsorption—indicates that specific organ systems (neurologic, autoimmune, biliary, and neoplastic) warrant focused post-marketing surveillance. While causality cannot be inferred solely from FAERS data, systematic signal detection of this nature can guide periodic label revision, strengthen risk communication with prescribers, and prioritize events for targeted follow-up in registries and observational studies. Clinically, these findings underscore the need for age- and comorbidity-conscious prescribing—particularly in elderly patients and those receiving antiplatelet therapy—structured early follow-up to detect “early-failure” reactions, and selective endoscopic or biochemical monitoring in high-risk groups. Collectively, these measures align with a learning health system framework, wherein real-world pharmacovigilance data continuously refine clinical practice and inform regulatory oversight.

In conclusion, Vonoprazan demonstrates a measurable risk of gastrointestinal adverse events in real-world clinical use and may also be associated with novel, previously unreported reactions. Despite the inherent limitations of passive reporting and incomplete data within the FAERS database, the findings of this study provide an initial evidence base for informed clinical application and ongoing pharmacovigilance. Clinicians should individualize Vonoprazan therapy according to patient age, comorbidities, indication, and concomitant medications, balancing therapeutic efficacy against potential long-term risks. Integration of pharmacovigilance insights into prescribing practice may enhance patient safety in real-world settings.

Although this study identified several potential adverse event signals from FAERS database, the inherent limitations of this passive reporting system may influence the precision and interpretation of results. First, FAERS depends on voluntary reports from healthcare professionals, patients, and manufacturers, resulting in underreporting and various reporting bias. Second, many FAERS reports lack essential clinical information, such as dosage, duration, comorbidities, and concomitant medications, limiting contextual interpretation and potentially signal detection. Third, because FAERS lacks denominator data on drug exposure, incidence rates and comparative risk assessments cannot be estimated, restricting interpretation to disproportionality rather than causal inference. Consequently, formal causality assessment frameworks such as the Naranjo algorithm and WHO-UMC criteria were not applied due to missing case-level information. Moreover, FAERS does not consistently capture dechallenge/rechallenge data, dose modifications, or adjudicated clinical evaluations required for definitive causal attribution. Therefore, the identified Preferred Terms (PTs) should be regarded as hypothesis-generating safety signals warranting prospective validation rather than confirmed drug toxicities. These limitations may lead to overestimation, underestimation, or spurious positive signals. Importantly, the observed disproportionality signals may also reflect reporting behavior—such as heightened awareness of gastrointestinal events after U. S. marketing—rather than true biological excess risk.

To mitigate these potential biases, several methodological safeguard were incorporated into the study design. First, four widely used disproportionality analysis methods were applied in parallel to cross-validate detected signals and enhance the robustness of results. Second, only statistically significant (*p* < 0.05) and directionally consistent signals across multiple algorithms were retained to ensure reliability and reproducibility. Third, identified signals were compared with publicly available FDA adverse reaction data to exclude previously recognized events and emphasize potential novel findings. Fourth, preliminary interpretations of selected signals were provided from clinical and pathophysiological perspectives to enhance interpretability. Overall, despite the intrinsic biases and data incompleteness of the FAERS database, the use of multi-method cross-validation and stringent screening criteria confers additional robustness to our findings. These findings provide a preliminary foundation for evaluating vonoprazan’s the safety profile and guiding clinical risk management, while further validation through prospective studies and mechanistic research remains warranted.

## 5 Conclusion

This study conducted a systematic pharmacovigilance analysis of vonoprazan- associated adverse events using FAERS data from 2023 to 2025. Gastrointestinal disorders represented the most frequently reported adverse events. Several serious adverse events exhibited elevated risks signals in specific demographic subgroups, particularly males, children, and the elderly. Sixteen unlabelled adverse events were detected, several with strong disproportionality signals suggestive of potential safety concerns. Most events occurredearly after treatment initiation, with earlier onset observed infemales. These findings generate hypotheses concerning demographic susceptibility, early-onset intolerance, and potential organ-system involvement. While not establishing causality, these results may aid clinicians in counseling high-risk patients (e.g., elderly individuals or those on antiplatelet therapy) and guide priorities for prospective monitoring and regulatory reassessment. Further pharmacoepidemiologic studies with validated clinical endpoints are warranted before any practice-changing recommendations can be made.

## Data Availability

Publicly available datasets were analyzed in this study. This data can be found at: https://fis.fda.gov/extensions/FPD-QDE-FAERS/FPD-QDE-FAERS.html.

## Footnotes

1 available at https://fis.fda.gov/extensions/FPD-QDE-FAERS/FPD-QDE-FAERS.html.

2 accessible at https://www.accessdata.fda.gov/scripts/cder/daf/index.cfm?event=overview.process&ApplNo=218710.
